# The Relationship between Long Noncoding RNA H19 Polymorphism and the Epidermal Growth Factor Receptor Phenotypes on the Clinicopathological Characteristics of Lung Adenocarcinoma

**DOI:** 10.3390/ijerph18062862

**Published:** 2021-03-11

**Authors:** Yao-Chen Wang, Shih-Ming Tsao, Yia-Ting Li, Chia-Yi Lee, Thomas Chang-Yao Tsao, Ming-Ju Hsieh, Shun-Fa Yang

**Affiliations:** 1 School of Medicine, Chung Shan Medical University, Taichung 402, Taiwan; wang5717@ms21.hinet.net (Y.-C.W.); tsmhwy@ms24.hinet.net (S.-M.T.); his885889@gmail.com (T.C.-Y.T.); 2 Department of Internal Medicine, Chung Shan Medical University Hospital, Taichung 402, Taiwan; 3 Division of Chest, Department of Internal Medicine, Chung Shan Medical University Hospital, Taichung 402, Taiwan; 4 Division of Respiratory Therapy, Department of Internal Medicine, Chung Shan Medical University Hospital, Taichung 402, Taiwan; miki2700@gmail.com; 5 Institute of Medicine, Chung Shan Medical University, Taichung 402, Taiwan; 6 Department of Ophthalmology, Show Chwan Memorial Hospital, Changhua 500, Taiwan; ao6u.3msn@hotmail.com; 7 Oral Cancer Research Center, Changhua Christian Hospital, Changhua 500, Taiwan; 8 Graduate Institute of Biomedical Sciences, China Medical University, Taichung 404, Taiwan; 9 Department of Medical Research, Chung Shan Medical University Hospital, Taichung 402, Taiwan

**Keywords:** long noncoding RNA H19, epithelial growth factor receptor, lung adenocarcinoma, single nucleotide polymorphism, tumor status

## Abstract

The aim of the current study is to investigate potential associations among Long Noncoding RNA (LncRNA) H19 single nucleotide polymorphism (SNP) and epidermal growth factor receptor (EGFR) phenotypes on the clinicopathological characteristics of lung adenocarcinoma (LADC). Five loci of LncRNA H19 SNPs (rs217727, rs2107425, rs2839698, rs3024270, and rs3741219) were genotyped by using TaqMan allelic discrimination in 223 LADC patients with wild-type EGFR phenotype and 323 LADC individuals with EGFR mutations. After the statistical analyses, patients with the EGFR mutation were related to a higher distribution frequency of rs217727 SNP CT heterozygote (*p* = 0.030), and the female population with EGFR mutation demonstrated a higher distribution frequency of rs217727 SNP CT heterozygote (*p* < 0.001) and rs2107425 CT heterozygote (*p* = 0.002). In addition, the presence of LncRNA H19 SNP rs217727 T allele (CT + TT) in patients with EGFR wild-type was associated to higher tumor T status (stage III or IV, *p* = 0.037) and poorer cell differentiation status (poor differentiation, *p* = 0.012) compared to those EGFR wild-type individuals with LncRNA H19 SNP rs217727 CC allele. Besides, a prominently higher tumor T status was found in subjects with LncRNA H19 SNP rs2107425 T allele (CT + TT) (stage III or IV, *p* = 0.007) compared to EGFR wild-type LADC individuals with LncRNA CC allele in EGFR wild-type patients. Our findings suggest that the presence of LncRNA H19 SNP rs217727 is related to the EGFR mutation in LADC patients, and the LncRNA H19 SNP rs217727 and rs2107425 are associated with progressed tumor status for LADC patients with EGFR wild-type.

## 1. Introduction

Lung adenocarcinoma (LADC) is the most common type of lung cancer throughout the world in both genders [1]. In previous epidemiological studies, the prevalence of LADC is more than 40 percent in a Chinese population [2] which also continuously outnumbered the squamous cell carcinoma for female population in Europe, North America and Oceania [3]. The known populations that are susceptible to LADC include older age, female sex, non-tobacco consumption and presence of certain genetic mutations [4,5]. In addition to conventional treatments for LADC, the new management like the target therapy and immunotherapy for LADC is under investigation in several experimental researches to resolve the drug-resistance issue [6].

There are several molecular biomarkers that could influence the clinical presentation of LADC and could be an alternative management for LADC in the future [7,8]. The different mutation of epidermal growth factor receptor (EGFR), a receptor belong to the ERBB family, would significantly affect the stage and treatment outcome in LADC which had been well-established in the previous studies [8,9]. The main EGFR mutation types are the L858R expression and Exon 19 in-frame deletion, which are associated with a better prognosis of LADC compared to the LADC with EGFR wild-type [10,11,12]. In addition to EGFR, the single nucleotide polymorphism (SNP) of other genes would also correlate to different clinical characteristics and treatment outcome of LADC [13,14]. In previous studies, both the endothelial nitric oxide synthase and carbonic anhydrase 9 were proven to alter the tumor grade of LADC but with different effect [15,16]. Besides, the concurrent presence of some SNPs of Aurora kinase A and EGFR mutation were related to an earlier tumor stage of LADC [17]. Consequently, there may exist other genes whose SNP, together with EGFR mutation, could contribute to different clinical characters and severities of LADC.

The Long Noncoding RNA (LncRNA) refers to a family of genetic sequences that do not contribute to the synthesis of protein but can regulate certain cellular functions like chromatin remodeling, cell cycle advancement, and neoplasm formation [18,19]. Among the LncRNA, the LncRNA H19 revealed certain associations to several types of cancers [20,21,22]. For the field of lung cancers, the high level of LncRNA H19 expression would lead to gefitinib resistance in non-small cell lung cancer, [23] and the presence of LncRNA H19 are associated with the proliferation of lung cancer cells [24]. Since some of the LncRNA could affect the susceptibility and clinical characters of EGFR mutation-related LADC [25,26], the SNP of LncRNA H19 may own some synergic effect with other somatic mutation like EGFR on the clinical course of LADC while the current evidence for this issue is inadequate.

Herein, the purpose of the current study is to evaluate the distribution of several LncRNA H19 SNPs in different EGFR phenotypes. Besides, the synergic effects of LncRNA H19 SNPs and EGFR phenotypes on the clinicopathological characteristics of LADC were also investigated.

## 2. Materials and Methods

### 2.1. Ethnic Declaration

The current study adhered to the declaration of Helsinki in 1964 and the corresponding late amendment. In addition, the current study was approved by both the Institutional Review Boards of Chung Shan Medical University Hospital (Project code: CS1-20144). The written informed consent was collected from all the participants in the current study, and it can be provided upon reasonable request.

### 2.2. Subject Selection

A prospective case-control study was conducted in the Chung Shan Medical University Hospital. The subjects which were diagnosed with LADC and followed up more than one year later in the Chung Shan Medical University Hospital were included and constituted as the study group. After the inclusion process, a total number of 546 patients with LADC were enrolled in the current study. The medical records of those participants were reviewed, and then the demographic data such as the age, gender and cigarette smoking status of each subject was recorded. For the tumor grading, the Tumor, Node, Metastasis (TNM) status and the tumor stages were clarified by two pulmonologists/oncologists, and the three extends of cell differentiation including well differentiated, moderately differentiated, and poorly differentiated were categorized via the rules of American Joint Committee on Cancer manual. For the analysis of genetic variants of both the LncRNA H19 and EGFR, venous blood drawing for all the patients were performed in the Chung Shan Medical University Hospital, and the venous blood was then preserved in the ethylenediaminetetraacetic acid-containing tube. After the preservation management, the venous blood sample was immediately centrifuged and preserved in the laboratory refrigerator at near −80 Celsius degrees for the subsequent analyses. In addition to the venous blood sample, the frozen specimen of LADC from each patient was collected after the incisional biopsy. If the genome of either LncRNA H19 or EGFR was degraded before the laboratory analysis procedure, the individual that provided that blood sample and associated frozen specimen were excluded from the current study.

### 2.3. Genomic DNA Extraction and EGFR Sequencing

The DNA extraction and sequencing process of EGFR was done according to previous experience [17]. Briefly, tumor tissue from frozen specimen was collected to extract the DNA via the application of QIAamp DNA Kit (Qiagen, Valencia, CA, USA) according to the manufacturer’s guide document. After the DNA genome was obtained, categories of EGFR, including the wild-type and mutation phenotype, were amplified and detected via the use of real-time polymerase chain reaction (PCR). After that, the DNA sequencing reaction was conducted by ABI PRISM 3130XL System (Applied Biosystems, Foster City, CA, USA). Two types of EGFR genomes were categorized after the above procedures: the EGFR wild-type and EGFR mutation type. To be more specific, EGFR mutation that was analyzed in the current study included phenotypes of both the L858R expression as well as Exon 19 in-frame deletion.

### 2.4. The Genotyping of LncRNA H19 SNPs via Real-Time PCR

Five SNPs of LncRNA H19, including the rs217727 (C/T), the rs2107425 (C/T), the rs2839698 (C/T), the rs3024270 (C/G), and the rs3741219 (A/G) were selected due to the related studies conducted earlier [21,22]. About the genotyping steps which use the same method as previous research [17], the DNA was firstly extracted from the leukocytes of the venous blood from each patient via the usage of the QIAamp DNA kits (Qiagen, Valencia, CA, USA) according to manufacturer’s guide instruction. After that, the allelic discrimination of the above LncRNA H19 SNPs including the rs217727 (C/T), the rs2107425 (C/T), the rs2839698 (C/T), the rs3024270 (C/G), and the rs3741219 (A/G) was evaluated via the use of ABI StepOne Real-Time PCR System (Applied Biosystems, Foster City, CA, USA). Then the results of Real-Time PCR were analyzed by SDS version 3.0 software (Applied Biosystems) via the TaqMan assay technique to enhance the integrity of Real-Time PCR. The minor allelic frequencies of the five LncRNA H19 SNPs were more than five percent after all the analysis processes.

### 2.5. Statistical Analysis

The SAS version 9.4 (SAS Institute Inc., Cary, NC, USA) was applied for all the statistical analyses of the current study. Descriptive analysis was used to show the demographic data, tumor stage including the TNM status, and tumor cell differentiation between the EGFR wild-type and EGFR mutation subtypes. Then the Mann-Whitney U test and Fisher’s exact test was used to survey the difference of both the categorical variables and continuous variables between the EGFR wild-type and mutation subtype. After that, the multiple logistic regression was applied to yield adjusted odds ratios (AOR) with correlated 95% confidence intervals (CI) for the five different LncRNA H19 SNPs’ distribution between the wild-type EGFR and EGFR mutation type after adjusting the age, gender and cigarette consumption. A *p* value that is less than 0.05 was regarded as statistically significant.

## 3. Results

### 3.1. Demographics of the Study Population

A total of 546 participants with LADC were recruited in the current study, with 223 EGFR wild-type patients and another 323 subjects with EGFR mutation. The mean age of EGFR wild-type group was 64.60 ± 12.22 and the EGFR mutation group aged 65.33 ± 12.62 and the age did not show significant value between the two groups (*p* = 0.501). However, the EGFR mutation group contained both more female individuals (*p* < 0.001) and more never-smokers (*p* < 0.001). About the characteristics of LADC, the tumor cells in EGFR mutation group revealed a higher rate of well differentiation (*p* < 0.001). The other clinical characters of the two groups are shown in Table 1.

### 3.2. Distribution Frequency of LncRNA H19 SNP in Different EGFR Phenotypes

About the genotype distribution of LncRNA H19 SNP between different EGFR phenotypes, the rs217727 SNP CT + TT heterozygote showed a higher distribution frequency in the EGFR mutation group (AOR: 1.46, 95% CI: 1.01–2.13, *p* = 0.047) which was mainly due to the higher distribution frequency of rs217727 SNP CT heterozygote in EGFR mutation group (AOR: 1.56, 95% CI: 1.05–2.34, *p* = 0.030). However, the distribution frequency of other LncRNA H19 SNPs were similar between the two groups (Table 2). In the subgroup analysis, the female population with EGFR mutation demonstrated a higher distribution frequency of rs217727 SNP CT heterozygote (AOR: 2.94, 95% CI: 1.62-5.33, *p* < 0.001), rs217727 SNP CT + TT heterozygote (AOR: 2.44, 95% CI: 1.44–4.15, *p* < 0.001), rs2107425 CT heterozygote (AOR: 2.62, 95% CI: 1.44-4.74, *p* = 0.002), and rs2107425 SNP CT + TT heterozygote (AOR: 2.14, 95% CI: 1.26–3.63, *p* = 0.005) (Table 3). On the other hand, the distribution frequency of rs2839698 SNP TT homozygote was significantly lower in female population with EGFR mutation (AOR: 0.42, 95% CI: 0.19–0.94, *p* = 0.035). (Table 3) Still, the distribution frequency of LncRNA H19 SNP was not affected by EGFR phenotypes in male population (Table 3).

### 3.3. Correlation of Distribution between LncRNA H19 and EGFR Phenotypes to the Clinicopathological Characteristics in LADC

Concerning the difference of clinicopathological characteristics in patients with specific LncRNA H19 SNP and EGFR phenotype distributions, the presence of LncRNA H19 SNP rs217727 T allele (CT + TT) in patients with EGFR wild-type was correlated to advanced tumor T status (stage III or IV, *p* = 0.037) and poorer cell differentiation status (poor differentiation, *p* = 0.012) compared to those EGFR wild-type individuals with LncRNA H19 SNP rs217727 CC allele. Besides, all the patients with LncRNA H19 SNP rs2107425 T allele (CT + TT) experienced a severe tumor T status (stage III or IV, *p* = 0.025), while the phenomenon was more prominent in patients with both EGFR wild-type and LncRNA H19 SNP rs2107425 CT + TT (stage III or IV, *p* = 0.007) compared to EGFR wild-type LADC individuals with LncRNA H19 SNP rs2107425 CC allele. The other combination of LncRNA H19 SNP and EGFR phenotype did not alter the clinicopathologic characteristics of LADC significantly which are illustrated in Table 4 and Table 5.

## 4. Discussion

In the current study, a higher distribution frequency of LncRNA H19 SNP rs217727 was found in the EGFR mutation LADC population. Besides, the female LADC patients with EGFR mutation phenotype showed different distribution of LncRNA H19 SNP rs217727, rs2107425 and rs2839698 compared to EGFR wild-type population. Moreover, the EGFR wild-type LADC subject with LncRNA H19 SNP rs217727 or rs2107425 was correlated to severe tumor grade, especially higher tumor T status.

There are various factors like SNPs that influence the disease severity and progression of LADC [10,12,13,14,27,28]. The presence of KRAS mutation would lead to poor prognosis [29] and the BRAF-inactivating mutations could result in the development of LADC. Besides, the EGFR is also a genetic prognostic factor for LADC that has been investigated in details and the EGFR phenotype has different effects on the clinical course of LADC [10,11]. In a previous study, the presence of EGFR mutation L858R expression showed a higher tumor disappearance rate of LADC than the EGFR wild-type [30]. Additionally, the EGFR mutation Exon 19 in-frame deletion revealed an intermediate to low pathologic grade tumors of LADC [31]. Moreover, the EGFR mutation can increase the treatment effectiveness for LADC in which the EGFR Exon 19 in-frame deletion mutation increase both the overall survival and progression-free survival rate of LADC receiving target therapy and chemotherapy [32], while several EGFR mutations were associated with a significantly longer overall survival of LADC [33]. In the present study, a higher distribution frequency of LncRNA H19 SNP rs217727 was found in the EGFR mutation LADC population and could be used in future clinical treatments. On the other hand, the LncRNA H19 is an imprinted gene that is located on the chromosome 11p15.5 of the maternal allele [20]. About its primary function, the LncRNA H19 exists in heart, skeletal muscle and breast and can modulate the cell proliferation, cell differentiation and the oncogenic effects for development of neoplasms [19,34,35]. The experimental study showed that an up-regulation process of LncRNA H19 was observed in cancer cell lines compared with the non-cancerous cell lines, which associated with an elevated invasive behavior in tumor cell [20]. In the clinical situation, several neoplasms including hepatocellular carcinoma, urothelial cell carcinoma and non-small cell lung cancer were found to be associated with the existence of LncRNA H19 SNP [18,36,37]. For the lung cancers, the LncRNA H19 SNP rs217727 would lead to higher incidence of lung squamous cell carcinoma and LADC [38].

In previous researches, the somatic receptor mutation to a cancer and the cancer-associated SNPs seldom analyze together. However, the recent research starts showing that the interaction between genetic variations like SNPs and somatic mutations [39], with some articles illustrating the cancer-related SNPs including TERT and HMGB1 being correlated to the chance of EGFR mutation and the clinical characters in EGFR-mutated LADC [16,25,40]. As a result, we speculate that the EGFR mutation is mixed with the individual’s whole genome and can be influenced by other cancer-related genes as well as its polymorphisms whether congenitally or progressively during lifetime. On the other hand, the LncRNA H19 SNP is associated with the clinical course of certain types of adenocarcinoma including the breast adenocarcinoma and gastric adenocarcinoma [41,42] and the effect of several LncRNA H19 SNP rs217727 on the susceptibility of LADC is also significant [38]. Moreover, the potential relationship of LncRNA on the EGFR pathway in several cancers had been proven [43,44]. Consequently, we hypothesize the LncRNA H19 SNPs may lead to an alteration of LADC-related genome including the EGFR pathway, and the co-existing of the LncRNA H19 SNPs and EGFR mutation may lead to a modification on the tumorigenesis pathway of LADC, which at least is partially supported by the results of the current study.

The different distribution frequency of LncRNA H19 SNP in EGFR mutation was found in the current study which has seldom been presented elsewhere. The previous review article showed that the LncRNA H19 SNP rs217727 was significantly higher in the oral squamous cell carcinoma and lung cancer [45]. In addition, the T/T genotype of LncRNA H19 SNP rs217727 had a higher risk of lung cancer development than those of C/C genotype [38]. The above evidences suggested the LncRNA H19 SNP rs217727 altered the expressions for different cancers which our study supported with the previous literatures. In the gender subgroup analysis of the current study, the female EGFR mutation population showed a higher distribution frequency of LncRNA H19 SNP rs217727 as well as rs2107425 but a lower distribution frequency of LncRNA H19 SNP rs2839698. The difference of LncRNA H19 SNP distribution between the two gender subgroups may be due to the existence of expression quantitative trait loci which lead to gender-difference expression of LncRNA inter chr4 3011 in mice [46] and the possible difference function of EGFR pathway between gender, which is found in the kidney mouse [47], may also cause such gender difference as the expression of LncRNA PACER in periodontitis [48]. Since the LncRNA H19 SNP rs217727 was related to lung cancer development [45] and LncRNA H19 SNP rs2107425 was correlated to shorter metastasis-free survival [49], it may be reasonable for the similar condition in the current study. On the other hand, the LncRNA H19 SNP rs2839698 was related to the lower risk of bladder cancer [50]. Maybe the LncRNA H19 SNP rs2839698 could serve as a protective factor for at least the two malignancies. Unlike the female population, the male population did not show any tendency of distribution frequency of LncRNA H19 SNP and the exact etiology for such difference needs further investigation to confirm.

Concerning the clinicopathological characteristics of LADC, PD-1 SNP, ANGPT1 SNP and HDAC9 SNP had been proven to influence the tumor progression significantly [51,52,53]. In the current study, nevertheless, the correlation of LncRNA H19 SNP rs217727 and rs2107425 to the tumor progression of LADC with EGFR wild-type has been observed which is a preliminary experience for this field. To be more specific, the LncRNA H19 SNP rs217727 was related to a larger tumor T status and poor cell differentiation of LADC while the LncRNA H19 SNP rs2107425 was associated with a larger tumor T status only. In a preceding research, the poor-differentiated tumor cells of LADC were found in the patients with poor prognosis [54]. As a result, the LncRNA H19 SNP rs217727 might be a more important prognostic factor for LADC with EGFR wild-type than LncRNA H19 SNP rs2107425 because it produces a double risk. Moreover, the LncRNA H19 SNP rs217727 also related to different distribution frequency of EGFR mutation in LADC prominently, thus it may be the most important confounder for LADC considering the synergic effect with EGFR phenotype. However, the presence of LncRNA H19 SNP rs2107425 on the tumor T status is significant even in the enrolled study population with different EGFR phenotype, which implies the influence of LncRNA H19 SNP rs2107425 on LADC disease course cannot be overlooked especially for those with EGFR wild-type.

About the demographic data between the different EGFR phenotypes, the distribution of gender, smoking condition and tumor cell differentiation status demonstrated significant difference between the two groups. The LADC patients with EGFR mutation often develop in female gender and never-smokers according to previous research [5] and the results of the current study further clarify these concepts. The higher rate of EGFR mutation may result from the effect of exposure to carcinogens like female steroid hormones in which the rate of EGFR mutation elevated with age in female population [55]. The higher rate of EGFR mutation in never-smokers was frequently observed in the Eastern Asian population [5] while the exact etiology needs further validation. The percentage of poor-differentiated cell of LADC with EGFR mutation in the current study was significantly lower in the current study compared to the LADC patients with EGFR wild-type, which also corresponded to a previous study conducted in the Eastern Asian population [56]. The pathophysiology for this finding still needs elucidation, and we speculated both the L858R expression and Exon 19 in-frame deletion EGFR mutations may contribute to functional loss of LADC and generally benign clinical characteristics since higher sensitivity to tyrosine kinase inhibitor and better overall survival were found in patients with such mutations [11,57].

There are still some limitations in the current study. First, the small study population may diminish the statistical power in the current study. Second, the lifestyle and daily activity were not recorded in the medical documents thus certain risk factors cannot be considered in the multiple logistic regressions. Third, due to the lack of the survival data, the impact between the H19 polymorphism and follow-up clinical data could not be performed. Moreover, the other EGFR phenotypes such as del2236-2250 and T790M were not enrolled in the analysis of the current study. Still, since the incidence of the above two EGFR phenotypes are relatively low, the influence of not including them might not disturb the results of the current study to a large extent.

## 5. Conclusions

In conclusion, the distribution frequency of LncRNA H19 SNP rs217727 CT is higher in patients with LADC and EGFR mutation. Moreover, both the existences of LncRNA H19 SNP rs217727 CT as well as rs2107425 CT are higher in female LADC subjects with EGFR mutation. Furthermore, the LncRNA H19 SNP rs217727 and rs2107425, especially the LncRNA H19 SNP rs217727, are correlated to an advanced tumor status for LADC individuals with EGFR wild-type. Further large prospective studies to evaluate the effect of LncRNA H19 SNP rs217727 or rs2107425 on the disease progression, therapeutic effectiveness and prognosis of EGFR wild-type LADC is mandatory.

## Figures and Tables

**Table 1:** Demographics and clinical characteristics of 546 patients in lung adenocarcinoma with EGFR mutation status.

Variable	EGFR Wild-Type (*N* = 223) *n* (%)	EGFR Mutation (*N* = 323) *n* (%)	*p* Value
Age			
Mean ± SD	64.60 ± 12.22	65.33 ± 12.62	0.501
Gender			
Male	137 (61.4%)	109 (33.7%)	<0.001
Female	86 (38.6%)	214 (66.3%)	
Cigarette smoking status			
Never-smoker	105 (47.1%)	260 (80.5%)	<0.001
Ever-smoker	118 (52.9%)	63 (19.5%)	
Stage			
I + II	50 (22.4%)	77 (23.8%)	0.700
III + IV	173 (77.6%)	246 (76.2%)	
Tumor T status			
T1 + T2	111 (49.8%)	174 (53.9%)	0.346
T3 + T4	112 (50.2%)	149 (46.1%)	
Lymph node status			
Negative	61 (27.4%)	90 (27.9%)	0.896
Positive	162 (72.6%)	233 (72.1%)	
Distant Metastasis			
Negative	99 (44.4%)	129 (39.9%)	0.299
Positive	124 (55.6%)	194 (60.1%)	
Cell differentiation			
Well	19 (8.5%)	35 (10.8%)	<0.001
Moderately	136 (61.0%)	251 (77.7%)	
Poorly	68 (30.5%)	37 (11.5%)	

*N*: number; SD: standard deviation.

**Table 2:** Distribution frequency of *H19* genotypes of patients with lung adenocarcinoma and multiple logistic regression analysis of EGFR mutation association.

Genotypes	Wild-Type (*N* = 223)	EGFR Mutation (*N* = 323)	AOR (95% CI)	*p* Value
rs217727				
CC	97 (43.5%)	125 (38.7%)	1.00	
CT	95 (42.6%)	154 (47.7%)	1.56 (1.05–2.34)	0.030
TT	31 (13.9%)	44 (13.6%)	1.20 (0.68–2.11)	0.543
CT + TT	126 (56.5%)	198 (61.3%)	1.46 (1.01–2.13)	0.047
rs2107425				
CC	81 (36.3%)	106 (32.8%)	1.00	
CT	101 (45.3%)	163 (50.5%)	1.46 (0.97–2.21)	0.071
TT	41 (18.4%)	54 (16.7%)	0.97 (0.57–1.65)	0.915
CT + TT	142 (63.7%)	217 (67.2%)	1.31 (0.89–1.92)	0.176
rs2839698				
CC	105 (47.1%)	157 (48.6%)	1.00	
CT	88 (39.5%)	140 (43.3%)	1.01 (0.68–1.49)	0.981
TT	30 (13.4%)	26 (8.1%)	0.53 (0.28–1.01)	0.061
CT + TT	118 (52.9%)	166 (51.4%)	0.88 (0.61–1.27)	0.492
rs3024270				
CC	60 (26.9%)	94 (29.1%)	1.00	
CG	108 (48.4%)	166 (51.4%)	0.95 (0.62–1.46)	0.811
GG	55 (24.7%)	63 (19.5%)	0.68 (0.41–1.15)	0.155
CG + GG	163 (73.1%)	229 (70.9%)	0.86 (0.57–1.29)	0.468
rs3741219				
AA	101 (45.3%)	154 (47.7%)	1.00	
AG	90 (40.4%)	135 (41.8%)	0.92 (0.62–1.36)	0.659
GG	32 (14.3%)	34 (10.5%)	0.63 (0.35–1.12)	0.116
AG + GG	122 (54.7%)	169 (52.3%)	0.84 (0.58–1.21)	0.351

The AORs with 95% CIs were estimated by multiple logistic regression models after controlling for age, gender and cigarette smoking status. *N*: number; AOR: adjusted odds ratio; CI: confidence interval.

**Table 3:** Distribution frequency of *H19* genotypes of 300 female patients with lung adenocarcinoma and multiple logistic regression analysis of EGFR mutation association.

Genotypes	Male (*N* = 246)			Female (*N* = 300)		
	Wild-Type (*N* = 137)	EGFR Mutation (*N* = 109)	AOR (95% CI)	Wild-Type (*N* = 86)	EGFR Mutation (*N* = 214)	AOR (95% CI)
rs217727						
CC	46 (33.6%)	40 (36.7%)	1.00	51 (59.3%)	85 (39.7%)	1.00
CT	73 (53.3%)	54 (49.5%)	0.85 (0.48–1.52)	22 (25.6%)	100 (46.7%)	2.94 (1.62–5.33) *^,a^
TT	18 (13.1%)	15 (13.8%)	0.87 (0.37–2.01)	13 (15.1%)	29 (13.6%)	1.54 (0.71–3.34)
CT + TT	91 (66.4%)	69 (63.3%)	0.86 (0.49–1.49)	35 (40.7%)	129 (60.3%)	2.44 (1.44–4.15) *^,b^
rs2107425						
CC	39 (28.5%)	36 (33.0%)	1.00	42 (48.8%)	70 (32.7%)	1.00
CT	75 (54.7%)	59 (54.1%)	0.82 (0.45–1.48)	26 (30.2%)	104 (48.6%)	2.62 (1.44–4.74) *^,c^
TT	23 (16.8%)	14 (12.8%)	0.57 (0.25–1.33)	18 (20.9%)	40 (18.7%)	1.46 (0.73–2.92)
CT + TT	98 (71.5%)	73 (67.0%)	0.76 (0.43–1.34)	44 (51.2%)	144 (67.3%)	2.14 (1.26–3.63) *^,d^
rs2839698						
CC	70 (51.1%)	58 (53.2%)	1.00	35 (40.7%)	99 (46.3%)	1.00
CT	52 (38.0%)	43 (39.4%)	1.04 (0.60–1.80)	36 (41.9%)	97 (45.3%)	0.96 (0.55–1.68)
TT	15 (10.9%)	8 (7.3%)	0.69 (0.26–1.80)	15 (17.4%)	18 (8.4%)	0.42 (0.19–0.94) *^,e^
CT + TT	67 (48.9%)	51 (46.8%)	0.96 (0.57–1.62)	51 (59.3%)	115 (53.7%)	0.80 (0.48–1.35)
rs3024270						
CC	40 (29.2%)	34 (31.2%)	1.00	20 (23.3%)	60 (28.0%)	1.00
CG	67 (48.9%)	58 (53.2%)	1.04 (0.57–1.90)	41 (47.7%)	108 (50.5%)	0.84 (0.45–1.59)
GG	30 (21.9%)	17 (15.6%)	0.80 (0.37–1.74)	25 (29.0%)	46 (21.5%)	0.58 (0.28–1.18)
CG + GG	97 (70.8%)	75 (68.8%)	0.97 (0.55–1.72)	66 (76.7%)	154 (72.0%)	0.74 (0.41–1.35)
rs3741219						
AA	68 (49.6%)	58 (53.2%)	1.00	33 (38.4%)	96 (44.9%)	1.00
AG	53 (38.7%)	41 (37.6%)	0.92 (0.53–1.61)	37 (43.0%)	94 (43.9%)	0.91 (0.52–1.59)
GG	16 (11.7%)	10 (9.2%)	0.82 (0.34–2.01)	16 (18.6%)	24 (11.2%)	0.51 (0.24–1.10)
AG + GG	69 (50.4%)	51 (46.8%)	0.90 (0.53–1.52)	53 (61.6%)	118 (55.1%)	0.79 (0.47–1.33)

The AORs with 95% CIs were estimated by multiple logistic regression models after controlling for age and cigarette smoking status. N: number; AOR: adjusted odds ratio; CI: confidence interval. * *p* < 0.05, ^a^ *p* < 0.001, ^b^ *p* = 0.001, ^c^ *p* = 0.002, ^d^ *p* = 0.005, ^e^ *p* = 0.035.

**Table 4:** Clinicopathologic characteristics of lung adenocarcinoma patients with EGFR mutation, stratified by polymorphic genotypes of H19 rs217727.

Variable	ALL (*N* = 546)			EGFR Wild-Type (*N* = 223)			EGFR Mutation (*N* = 323)		
	CC (*N* = 222)	CT + TT (*N* = 324)	*p* Value	CC (*N* = 97)	CT + TT (*N* = 126)	*p* Value	CC (*N* = 125)	CT + TT (*N* = 198)	*p* Value
Stages									
I + II	52 (23.4%)	75 (23.1%)	0.940	24 (24.7%)	26 (20.6%)	0.466	28 (22.4%)	49 (24.7%)	0.630
III + IV	170 (76.6%)	249 (76.9%)		73 (75.3%)	100 (79.4%)		97 (77.6%)	149 (75.3%)	
Tumor T status									
T1 + T2	125 (56.3%)	160 (49.4%)	0.112	56 (57.7%)	55 (43.7%)	0.037	69 (55.2%)	105 (53.0%)	0.703
T3 + T4	97 (43.7%)	164 (50.6%)		41 (42.3%)	71 (56.3%)		56 (44.8%)	93 (47.0%)	
Lymph node status									
Negative	62 (27.9%)	89 (27.5%)	0.906	27 (27.8%)	34 (27.0%)	0.888	35 (28.0%)	55 (27.8%)	0.965
Positive	160 (72.1%)	235 (72.5%)		70 (72.2%)	92 (73.0%)		90 (72.0%)	143 (72.2%)	
Distant metastasis									
Negative	100 (45.0%)	128 (39.5%)	0.197	48 (49.5%)	51 (40.5%)	0.180	52 (41.6%)	77 (38.9%)	0.628
Positive	122 (55.0%)	196 (60.5%)		49 (50.5%)	75 (59.5%)		73 (58.4%)	121 (61.1%)	
Cell differentiation									
Well/Moderately	186 (83.8%)	255 (78.7%)	0.139	76 (78.4%)	79 (62.7%)	0.012	110 (88.0%)	176 (88.9%)	0.807
Poorly	36 (16.2%)	69 (21.3%)		21 (21.6%)	47 (37.3%)		15 (12.0%)	22 (11.1%)	

*N*: number.

**Table 5:** Clinicopathologic characteristics of lung adenocarcinoma patients with EGFR mutation, stratified by polymorphic genotypes of H19 rs2107425.

Variable	ALL (*N* = 546)			EGFR Wild-Type (*N* = 223)			EGFR Mutation (*N* = 323)		
	CC (*N* = 187)	CT + TT (*N* = 359)	*p* Value	CC (*N* = 81)	CT + TT (*N* = 142)	*p* Value	CC (*N* = 106)	CT + TT (*N* = 217)	*p* Value
stages									
I + II	42 (22.5%)	85 (23.7%)	0.749	20 (24.7%)	30 (21.1%)	0.539	22 (20.8%)	55 (25.3%)	0.363
III + IV	145 (77.5%)	274 (76.3%)		61 (75.3%)	112 (78.9%)		84 (79.2%)	162 (74.7%)	
Tumor T status									
T1 + T2	110 (58.8%)	175 (48.7%)	0.025	50 (61.7%)	61 (43.0%)	0.007	60 (56.6%)	114 (52.5%)	0.491
T3 + T4	77 (41.2%)	184 (51.3%)		31 (38.3%)	81 (57.0%)		46 (43.4%)	103 (47.5%)	
Lymph node status									
Negative	48 (25.7%)	103 (28.7%)	0.454	20 (24.7%)	41 (28.9%)	0.500	28 (26.4%)	62 (28.6%)	0.685
Positive	139 (74.3%)	256 (71.3%)		61 (75.3%)	101 (71.1%)		78 (73.6%)	155 (71.4%)	
Distant metastasis									
Negative	86 (46.0%)	142 (39.6%)	0.148	41 (50.6%)	58 (40.8%)	0.158	45 (42.5%)	84 (38.7%)	0.519
Positive	101 (54.0%)	217 (60.4%)		40 (49.4%)	84 (59.2%)		61 (57.5%)	133 (61.3%)	
Cell differentiation									
Well/Moderately	153 (81.8%)	288 (80.2%)	0.654	61 (75.3%)	94 (66.2%)	0.155	92 (86.8%)	194 (89.4%)	0.489
Poorly	34 (18.2%)	71 (19.8%)		20 (24.7%)	48 (33.8%)		14 (13.2%)	23 (10.6%)	

*N*: number.

## Data Availability

Not applicable.
